# A Survey of Soft Computing Approaches in Biomedical Imaging

**DOI:** 10.1155/2021/1563844

**Published:** 2021-08-02

**Authors:** Manju Devi, Sukhdip Singh, Shailendra Tiwari, Subhash Chandra Patel, Melkamu Teshome Ayana

**Affiliations:** ^1^Deenbandhu Chhotu Ram University of Science and Technology, Murthal, Sonipat, Haryana, India; ^2^Thapar Institute of Engineering and Technology (TIET), Patiala, Punjab, India; ^3^School of Computer Science and Engineering, VIT Bhopal University, Bhopal, India; ^4^Arba Minch University, Arba Minch, Ethiopia

## Abstract

Medical imaging is an essential technique for the diagnosis and treatment of diseases in modern clinics. Soft computing plays a major role in the recent advances in medical imaging. It handles uncertainties and improves the qualities of an image. Until now, various soft computing approaches have been proposed for medical applications. This paper discusses various medical imaging modalities and presents a short review of soft computing approaches such as fuzzy logic, artificial neural network, genetic algorithm, machine learning, and deep learning. We also studied and compared each approach used for other imaging modalities based on the certain parameter used for the system evaluation. Finally, based on comparative analysis, the possible research strategies for further development are proposed. As far as we know, no previous work examined this issue.

## 1. Introduction

**Figure 1:**
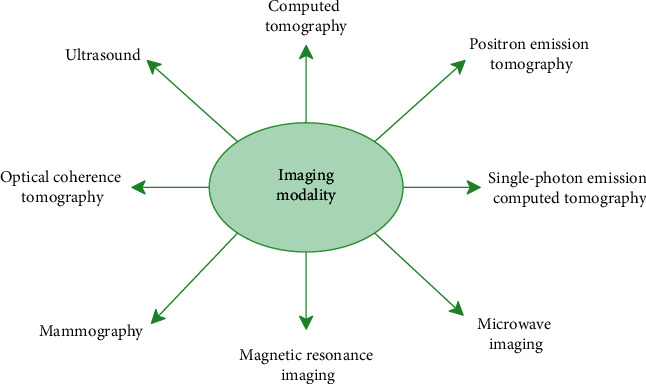
Imaging modalities diagram.

Medical imaging offers a noninvasive technique to look at the practical and structural information of internal organs. Currently, in medical imaging, a wide number of different image modalities are used. These modalities enable the radiologist to acquire a perfect spatial resolution in a noninvasive manner, typically providing the three-dimensional view of the anatomical and functional behaviour of the internal structure of human bodies like the heart, kidney, liver, and spleen. More tests are to be conducted to detect changes in the heart rate, blood supply, chemical composition, and blood absorption these days because of the usefulness of imaging devices. There are several medical imaging modalities like “computed tomography (CT), Ultrasound, Positron Emission Tomography (PET), Single-Photon Emission Computed Tomography (SPECT), Optical Coherence Tomography (OCT), Mammography, Magnetic Resonance Imaging (MRI), and Microwave Imaging” and so forth, as illustrated in Figure 1.

### 1.1. Computed Tomography (CT)

CT is a method of measuring an object's cross-sections using a series of X-ray measurements taken around the body from various angles. The CT system is typically calculated by the X-ray source, detector, and scanning direction. It is the most popular modality used in clinical diagnosis to detect abnormalities such as cancer, tumors, or organ deficiencies [1, 2]. CT has become an effective method for supplementing X-rays and ultrasonography in medical imaging. CT eliminates the superimposition of pictures of an object outside the field of interest. The core principles of the X-ray computed tomography involve X-ray generation, processing, identification, digitization, and image reconstruction which can be used to represent and analyze objects without physical harm, which provides several advantages in relevant areas.

First, it illustrates a standard procedure of a medical CT scan; the patient is to lie on a hospital bed. The CT system then moves the patient to find the right location for the scanning. Then it activates the X-ray source and rotates around the patient. In the meantime, X-ray detectors are located in a field on the other side. While the X-ray source is spinning, sensors record the scanned patient's one-dimensional projection. The data from the projection are recorded during this process. If the camera system is done spinning, all the one-dimensional projections are spliced, making a picture that looks like multiple superimposed sinusoidal pictures. This image is called a sinogram and is the most common source of raw CT scanning data for organizations. Finally, the sinogram is implemented using an image reconstruction algorithm, generating a tomography representation of the patient body.

### 1.2. Positron Emission Tomography (PET)

PET is a type of nuclear imaging method which produces a 3D image of the biological process in the human body by measuring the radiation emitted by photons. In 1975, the first commercial PET scanner was introduced, and in the 1990s it was used in clinics regularly [3]. PET is the latest imaging method with a wide variety of medical uses. Although such techniques are initially developed for use in studying the brain's functional features, they are now commonly used in numerous clinical applications, including cancer detection, cardiac disease, and neurological disorders. PET medical imaging can be subcategorized in functional and anatomical imagery; the first obtains anatomical images of a body, and the second produces images of physiological activity. Constant growth in algorithm development is taking place over a decade, intending to analyze 2D and 3D images derived from a PET scanner.

### 1.3. Single-Photon Emission Computed Tomography (SPECT)

It is a standard method of nuclear imaging, increasing its role in investigating and managing a wide variety of neurological disorders [4]. Radioactive elements and a purpose-built gamma camera are used to create three-dimensional images of the organs inside. This form of imaging offers physicians a noninvasive way to measure the health of certain parts of a body, most notably the heart, brain, and bones. What separates SPECT scans from other imaging techniques is that the scan will reveal how well those organs operate. For example, SPECT scan images can help determine the location of seizures in people with epilepsy and evaluate if there is enough blood flow to various parts of the brain.

*Purpose of Test*. SPECT scans may be used for many purposes, which is why, in most hospitals, clinics, and imaging centers, they are readily accessible. Some of the reasons your doctor may decide to order this test involve the concern or need to monitor

- brain and neurological conditions
- cardiac conditions
- bone disorders

Like other nuclear scans, SPECT uses radioactive tracers, carrier molecules bound with radioactive atoms, to identify, diagnose, and treat various diseases. Various tracers perform multiple tasks based on the symptoms or condition being tested, and the doctor selects the best tracer for you. The SPECT machine is an extensive circular system containing a camera detecting the radioactive tracer that absorbs the body. During the scan, you lie on a table, while the SPECT device rotates around you. The SPECT computer captures the internal organs and other structures. The images are sent to a machine that produces 3D images of your body using the details. Since the SPECT scan uses a low dose of radiation, if you have any questions about the risk of exposure, speak with your doctor. Usage of this imaging approach was not associated with any long-term health risks.

### 1.4. Magnetic Resonance Imaging (MRI)

MRI is a noninvasive biomedical imaging method that uses a powerful periodic magnetic field to produce radio waves that can be detected and used in the MRI scanner to create two- and three-dimensional pictures of a living object. It is used to create images of physiological processes, organs, and tissues within the body. MRIs are especially used to represent the body's nonbony or soft tissue sections. The most significant difference compared to CT scans is that it uses ionizing X-ray radiation. In contrast with X-ray and CT scans, knee and shoulder injuries are shown with greater precision than MRI scans. MRI scans would be used in the brain to differentiate between grey matter and white matter, and that in effect helps to understand tumor and aneurysms. The Open Access Sequence of Imaging Studies project has collected neuroimagery datasets containing more than 2000 MRI lessons for biomedical imaging researchers.

### 1.5. Optical Coherence Tomography (OCT)

OCT is a noninvasive imaging method that acquires high-resolution cross-sectional 10-micron pictures of the retina and anterior segments. This method uses low-coherence light to obtain two- and three-dimensional micrometre-resolution images from within the biological tissues. It is used mainly to diagnose eye issues by offering a cross-sectional illustration of the retina, enabling the doctor to see the whole layer. It allows mapping of the coating and calculating the thickness, which is helpful for the treatment.

### 1.6. Ultrasound (US)

US imagery includes exposing a body part with high-frequency sound waves to create an image of the body's inside. Because images are taken in real time, they can reveal the structure and activity of the body's internal organs and the blood moving through the blood vessels. The US imaging method uses sound waves of high frequency to create visual representations of internal organs, tissues, and blood supply. It is the procedure that is mostly included in pregnancy to control the fetus. It is often used for stomach, renal, and thyroid scans and is not usually used for the imaging of air-containing bones or tissues, including lungs. The advantage of using the US is that it is quick without any radiation.

Among the medical imaging modalities, “Ultrasound (US), computed tomography (CT), Magnetic Resonance Imaging (MRI), and Positron Emission Tomography (PET)” imaging have been of great importance in several areas of research. Both anatomical and functional imaging modalities are incredibly relevant in many medical fields, such as “computer-aided diagnosis, pathology follow-up, patient monitoring, and therapy (radiotherapy, chemotherapy, etc.).” In all these kinds of clinical application, computer assistance plays a significant role. Due to the technology of medical image analysis that has overgrown over the last decades, there have been significant facilities for clinical examinations.

## 2. Overview

**Figure 2:**
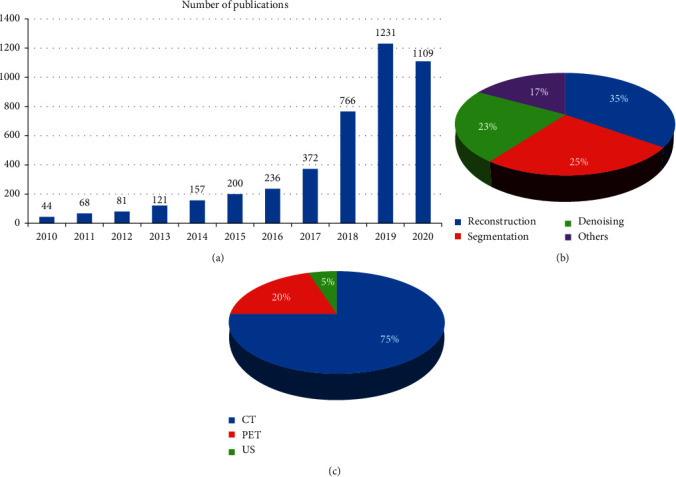
Publication of papers per-year relevant to biomedical application work using SC techniques over the period of 2010–2020 (a), distribution per paper objective (b), and distribution per modality form (c).

It is easy to see that researchers have found soft computing (SC) approaches by conducting a search on the PubMed website using “biomedical image application using SC techniques” as the keyword and choosing “article” as the type of text, since the number of articles has increased significantly since 2010 (Figure 2), reaching more than 1231 publications in 2019. This growth is largely explained by the rise in SC approaches (Figure 2). After that, the sources to be consulted were chosen. Four well-established databases, Web of Science, Google Scholar, PubMed, and Springer, have been used. A combination of the key terms were included in the search strings: (genetic algorithm) OR (machine learning) OR (deep learning) OR (fuzzy logic), CT, PET, Reconstruction, and Segmentation. By combining similar entries, the findings were scanned. They were then first screened with titles and abstracts based on them. After selecting the papers of interest, the main objective of the work, the anatomical interest, the methodology used, the evaluation metrics, and the attributes of the dataset used throughout the experiments, the main results and any other relevant details were carefully read in order to extract them. These data have been organized, and the current paper has been prepared.

It should be noted that most articles deal with a reconstruction issue, followed by segmentation and then denoising. Other forms of issues have been given less consideration. With regard to the data used, the CT prevalence is very high, followed by PET and then the US. The per-year evolution of the papers published is shown in Figure 2.

## 3. Soft Computing

Soft computing (SC) is introduced in medical imaging because it is an efficient method to deal with the uncertainties inherent in acquired image data [5]. SC methods are also used in the fields such as scientific study, medical science, management, and engineering. The inspiration for soft computing is to obtain artificial intelligence by replicating the human brain's thinking ability to solve the ambiguity of complex real-world problems. SC may be a fusion of computational methods and biological methods, which give efficient strategies for a more dynamic, skillful, and optimal solution. Lotfi A. Zadeh presented the idea of SC in the 1965s [6]. In this review paper, our focus is on core soft computing method such as “fuzzy logic, artificial neural network, and genetic algorithm” [5, 7].

**Figure 3:**
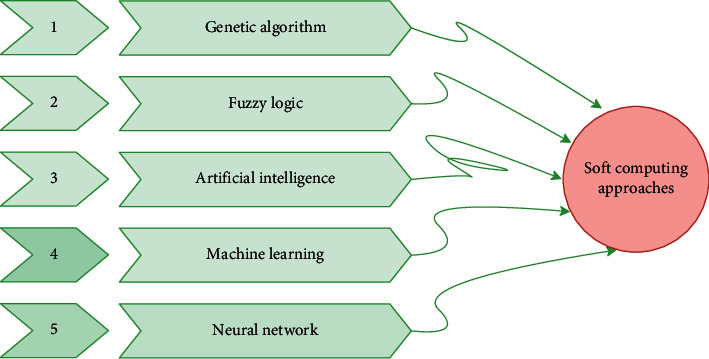
Soft computing approaches.

In comparison to hard computing, SC approaches accommodate imprecision, ambiguity, partial truth, and estimations. Acting flexibly with their roles makes them more efficient. Due to its adaptive nature and accuracy, the soft computing method is mainly used and preferred by researchers. It also has the benefits of cost-effectiveness, good efficiency, and robust solutions to complex problems. Many SC approaches are discussed in Figure 3.

### 3.1. Genetic Algorithm

The genetic algorithm (GA) method is motivated by the idea of biological evolution introduced by Darwin [8]. GA was investigated in this study for image reconstruction, image denoising, segmentation, image enhancement, and visualization. “GA is a technique widely used to overcome search & optimization problem for both restricted and unrestricted biological evolution-based data like mutation, crossover, and selection” [9].

The genetic algorithm can solve nondeterministic polynomial-time hardness (NP-Hard) problems that are impossible to solve in real time. With the application of the GA, we overcome complex problems quickly, which cannot be solved in mathematics. It is a heuristic or randomized search process that includes an initial solution set and produces a solution to the problem in an efficient and effective way. The preceding example of a person who wants to spend some cash in the bank is an easy way to understand these techniques. We understand that different banks have various policies and schemes are available. Their private interests are how much to invest in the fund so maximum profits can be made. How can he support and benefit from investing in the bank? These conditions can be solved by the “Evolutional Computing” method such as GA.

#### 3.1.1. A Survey of Genetic Algorithm

P. Lihue (2005) [10] developed a genetic algorithm for ECT image reconstruction. Algorithm initialization was based on linear back projection result. It is used to optimize the threshold and the minimum and maximum grey value for the image. The author stated that it is efficient and capable of reconstructing a high-quality image. The genetic algorithm-based method converges quickly with a small number of iterations.

Gouicem et al. [11] integrated the fuzzy penalty (FP) function and GA optimization for penalized-likelihood image reconstruction. The image was reconstructed from few projections in computed tomography. On the synthetic and real image datasets, this approach was tested and validated. It calculates speedily to a low noisy solution, even if the number of iterations is high, and provides a global estimation of finding object parameters, not a local one as in classical algorithms like a gradient.

DCruz et al. [12] proposed a system for detecting lung cancer while using the neural network and genetic algorithm Backpropagation. In this paper, classification was performed using Neural Network Backpropagation which would classify as normal or abnormal the digital X-ray, CT images, MRIs, and so forth. The normal condition is that which is characteristic of a healthy patient. For the study of the feature, the abnormal image will be considered further. The genetic algorithm can be used for adaptive analysis to extract and assign characteristics based on the fitness of the extracted factors. The features selected would be further classified as cancerous or noncancerous for images previously classified as abnormal. This method would then help to make an informed judgment on the status of the patient.

Liuet al. [13] presented a network evolution method that relies on the GA which checks for the most suitable genes to improve the structure of the networks. We accelerate the evolutionary process through a greedy discovery approach based on experience and transfer learning. A GA-based approach was proposed for automatically denouncing medical image CNN structures. We test and demonstrate EvoNet on a perfusion CT dataset.

Bahadure et al. [14] proposed segmentation techniques to improve tumor detection efficiency and computational efficiency; the GA is used for automated tumor stage classification. The choice in the classification stage shall be based on the extraction of the relevant features and the calculation of the area. The comparative approach is developed to compare four watersheds, FCM, DCT, and BWT-based segmentation techniques, and the highest is chosen by evaluating the segmentation score. The practical products of the proposed approach are evaluated and validated based on the segmentation ranking, accuracy, sensitivity, specificity, and dice similarity index coefficient for development and quality evaluation on MRI brain images.

A brief review of the work done by many researchers in the field of the biomedical image using genetic algorithm is summarized in Table 1, including a brief description of the modality, application, software used, and parameter evaluation.

### 3.2. Fuzzy Logic

In mathematics and engineering, fuzzy logic was first introduced by Zadeh in the mid-1965 [6]. A prosperous, diverse field of research [19] is the use of fuzzy approaches in image processing. Fuzzy-based approaches have already been used in various image processing areas, like filtering, interpolation [6], morphology, and segmentation. The fuzzy-based approach has already been used and has many practical applications. The iterative technique presented by Mondal et al. [20] using fuzzy potential function effectively reduces noise without affecting the image feature reconstructed.

#### 3.2.1. A Survey of Fuzzy Logic

Mondal and Rajan [20] presented a fuzzy-based method for iterative image reconstruction in Emission Tomography (ET). In this, two simple operations, fuzzy filtering and fuzzy smoothing, are performed. Fuzzy filtering is used for reconstruction to identify edges, while fuzzy smoothing is used for penalizing only those pixels for which the edges are missing in the nearest neighborhood. These operations are performed iteratively until appropriate convergence is achieved.

Bose [21] developed image segmentation techniques using fuzzy-based artificial bee colony (FABC). In that research, the author has combined the fuzzy c-means (FCM) and artificial bee colony (ABC) optimization to search for better cluster century. The proposed method FABC is more reliable than other optimization approaches like GA and PSO (particle swarm optimization). The experiment performed on grayscale images includes some synthetic medical and texture images. The proposed method has the advantages of fast convergence and low computational cost.

Debas et al. [22] developed an optimized Fuzzy Inference System (FIS) image reconstruction method to be implemented in capacitance tomography systems. The proposed model yields more precise solutions than other explicit methods but without increasing the computational costs. The process of image reconstruction, called “single-stage fuzzy,” offers improved time and resolution image reconstruction, making it an appealing model for ECT where real-time imagery is used. The accuracy and computational cost of the proposed approach make it a suitable method for the reconstruction of ECT structures.

Kala and Deepa [23] preserved the useful data; the suggested adaptive fuzzy hexagonal bilateral filter eliminates the Gaussian noise. The local and global evaluation metrics are used to create the fuzzy hexagonal membership function. The recommended method combines the median filter and the bilateral filter in an adaptive way. The bilateral filter is often used to retain the edges by smoothing the noise in the MRI image and by using a local filter to maintain the edges and obtain structural information. The proposed approach and the existing approach performed a series of experiments on synthetic and clinical brain MRI data at various noise levels. The outcome demonstrates that the proposed method restores the image to improved quality of the image which can be used for the diagnostic purpose well at both low and high Gaussian noise densities.

In Ghasemi et al.'s work [24], for medical image classification, a robust sparse representation is presented based on the adaptive type-2 fuzzy learning (T2-FDL) method. In the current procedure, sparse coding and dictionary learning method are iteratively performed until a near-optimum dictionary is produced. Two open-access brain tumor MRI databases, “REMBRANDT and TCGA-LGG,” from the Cancer Imaging Archive (TCIA), are used to conduct the experiments. The research findings of a classification task for brain tumors indicate that the implemented T2-FDL approach can effectively mitigate the adverse impacts of ambiguity in images data. The outcomes show the performance of the T2-FDL in terms of accuracy, specificity, and sensitivity compared to other relevant classification methods in the literature.

Table 2 summarizes the work done by many researchers who use fuzzy logic methods in different biomedical applications.

### 3.3. Artificial Intelligence

**Figure 4:**
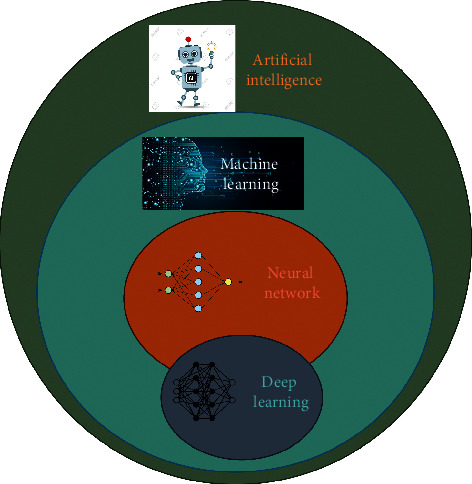
Artificial intelligence.

The term “artificial intelligence” was first used by mathematician John McCarthy, commonly known as AI's father, in 1956 to describe machines that do things that people would call smart [29]. “Artificial intelligence” (AI) refers to the development of computer systems that typically allow human intelligence to perform tasks. There are two paradigm shifts: (1) AI replaces people in problem-solving; (2) AI also replaces the traditional computational science and engineering algorithms. Such artificial intelligence systems are being created to improve medical image reconstruction, quality assurance, computer-aided detection, noise reduction, computer-aided classification, segmentation, and radio genomics. Machine learning, an AI subset, includes training techniques that perform work by learning from patterns and features derived from data. A neural network that initially comes from brain biology, passing information between the nodes called as artificial neurons is one approach employed in machine learning. Deep learning (DL) is a machine learning class defined by the use of DNN with various layers of mathematical concepts to do all the functions needed; that is, DL is a subset of machine learning, as shown in Figure 4, and ML is a subset of AI. We believe that AI is a new way to improve healthcare and achieve better outcomes at lower costs. So, AI is an excellent tool to build the future of imaging.

### 3.4. Machine Learning

Machine learning came into being in the late eighties and early nineties. Machine learning was coined by Arthur Samuel in 1959 [30]. This is a part of artificial intelligence, which allows the machine to behave and make data-driven decisions to achieve specific tasks. These programs are designed based on specific algorithms to learn and evolve themselves when exposed to the new data over time. In the past few years, the ML method has been used for image reconstruction, segmentation, classification, recognition of body organs from medical images, and so forth.

#### 3.4.1. A Review of Machine Learning

Pelt and Batenburg [31] introduced a new reconstruction technique in this research to resolve minimal data problems. In that research, the authors used the algebraic method to consider a two-problem high cost of computation and the necessary prior information which restrict the kinds of images which can be reconstructed. Neural Network Filtered Back Projection (NN-FBP) has been developed to solve these problems by the authors. If the accuracy of the proposed method depends on hidden layers, then reconstruction quality is not adequate. The NN-FBP can provide a more significant reconstruction than FBP and the computational complexity of NN-FBP compared to the algebraic method is low. But this paper restricts tomographic images by only two-dimensional parallel beams and trains too many hidden layers is a complex and time-consuming task.

The authors presented a new Penalized Weighted Least Squares (PWLS) approach for the LDCT reconstruction method that inhibits regularization following a prelearned square transform PWLS-ST and even involved the Union of Learned Transform (PWLS-ULTRA). The suggested method clusters the voxels into various classes and captures features such as bones, individual soft tissue, and edges. Experimental findings with Zheng et al. 2D and 3D axial CT IEEE Trans Med Imaging Page 17. Zheng et al. [34] demonstrate the experimental results of 2D and 3D axial CT tested over the XCAT phantom, 3D helical chest, and abdomen scans that indicate that the proposed methods include high-quality image reconstructions compared to traditional approaches like FBP or PWLS reconstruction with a nonadaptive edge-preserving regularization for both normal and low-dose levels. Compared with PWLS-ST, the ULTRA device provides a better transform system that allows significant reconstruction of different bones, individual soft tissues, and edges. The authors proposed an efficient iterative PWLS cost algorithm that would alternate between sparse coding and clustering step.

Li et al. [32] presented a novel Supervised-Unsupervised (SUPER) reconstruction framework for LDCT image reconstruction. It combined the advantages of supervised learning algorithms and transformed (unsupervised) learning-based techniques like PWLS-ULTRA that require detailed image-adaptive clustering. The SUPER model consists of multiple layers, one of which involves a deep network learned in a supervised way and an unsupervised iterative approach involving image-adaptive components. The SUPER reconstruction method is greedily known from the training data. This study proposed a new paradigm for LDCT image reconstruction, dubbed Supervised-Unsupervised (SUPER) learning. The structure allows various types of large data as a regularization to be leveraged effectively for CT reconstructions.

In Lee et al.'s work [33], the efficiency of processing CT projection data sinogram was calculated using a DL method for recognition of human anatomy and the detection of pathology. The authors developed a modified CNN called SinoNet, configured for sinogram analysis, and illustrated its ability by evaluating its output with previous CNN-based systems using reconstructed CT images. A square coevolutionary flap in the initial convolution layer was replaced by different rectangle convolution layer flaps of various sizes, including large and heightwise flaps. SinoNet's custom architecture has substantially better performance in human body recognition and ICH identification than sinogram's trained Inception-v3 models because of the sampling density. The outcomes indicate how nonsquare filters can enable systems to know the interactions among projection views and detector pixels through sinusoidal curves and extract salient classification aspects from the sinogram domain, a role those human experts find hard to understand. This method accelerates edge computing by allowing the rapid identification of the essential findings through actual data even without the time-consuming process of image reconstruction. This might enable us to build simpler scanner devices to detect critical results directly via SinoNet alone.

Table 3 lists some of the papers relevant to machine learning and its application in biomedical imaging.

### 3.5. Neural Network

Neural networks have gained high popularity in recent years, specifically for a method called deep learning, which uses extensive, complex neural networks [39]. AI techniques like deep learning and neural network have created a novel framework with novel approaches in inverse problems which could change the area.

#### 3.5.1. A Survey of Neural Network

Kartheeswarn [40] developed a sequential and parallel data decomposition technique based on PSO-ANN (particle swarm optimization with artificial neural networks). Generally, ANN training takes a long time; therefore, the author decomposes the dataset into a subset and assigned the weight of each subset optimized by PSO. Then, the training time is reduced using a similar strategy. However, the sequential approach consumes more training time.

Souza et al. [41] presented an algorithm to perform automatic CXR lung segmentation. It is used to solve the reconstruction of the “closed” lung areas due to pulmonary anomalies. The proposed approach uses two DNN convolution methods, and the proposed work has four stages named image acquisition, initial segmentation, reconstruction, and final segmentation. This approach was tested on 138 chest X-ray images from the tuberculosis prevention strategy of Montgomery County and obtained average sensitivity, specificity, accuracy, dice coefficient, and Jaccard index. Thus, dense abnormality in chest X-rays is being solved effectively in the lung segmentation system by executing a reconstruction step based on a DNN model.

Chan et al. [42] proposed the first efficient and convergent INN framework, Momentum-Net, by generalizing a block-wise MBIR approach using momentum and NN-regression majorizers. Momentum-Net uses momentum terminology in estimation components for fast MBIR and noniterative MBIR components for every layer via majorizers. Every other layer of Momentum-Net comprises three core components: image refinement, estimation, and MBIR. It ensures that they converge to a fixed point, under two asymptomatic conditions, for specific nonconvex MBIR variables and convex optimal sets. Also, a regularization parameter selection method based on the statistical radius of major matrices is suggested to understand the data-fit differences between training and testing sets. As a result, it achieves a quicker and more effective MBIR than traditional CNNs. Wu et al. [40] presented a deep CNN for CT image reconstruction. This study aims to decrease the memory and time usage of CT reconstruction network training to make it realistic for new processors while preserving the quality of the images reconstructed. The authors used DeepUNet here as CNN and implemented separate quadratic substitute with aggregate data fidelity subsets to solve the local-minimum problem of greedy learning to avoid simple local minima and obtain good image quality. This approach obtains better performance than iterative reconstruction based on total variation and dictionary learning for both two-dimensional and three-dimensional issues.

Table 4 summarizes a brief assessment of the work of various researchers in the field of biomedical image processing utilizing neural networks.

### 3.6. Generative Adversarial Network (GAN)

GAN, presented by Ian Goodfellow et al. in 2014 [50], is a class of AI algorithms commonly used in ML. A standard GAN structure comprises two neural networks contesting each other. The GAN system produces target data, and the other tries to separate it from ground reality. During this method, the efficiency of these two networks is enhanced continuously. The discriminator network makes GAN handle too complex data generation issues compared to traditional simple neural networks. It is commonly utilized in image processing problems due to its ability to produce data. It has significant benefits in image synthesis, semantic image processing, and design transfer over other networks. GAN has a generator network for generating a clean image from a reconstructed image from low-dose/sparse view/limited-angle data and a discriminator network for evaluating the generated image. GAN is structured as a generative model to create new data based on the information given rather than deleting or extracting data [51].

#### 3.6.1. A Survey of Generative Adversarial Network

In Pathak et al.'s work [52], the authors presented a new integrated low-dose CT reconstruction algorithm. This approach uses the “Global Dictionary-based Statistical Iterative Reconstruction (GDSIR) and Adaptive Dictionary-based Statistical Iterative Reconstruction (ADSIR)” method. In this situation, dictionary (D) is predetermined, and GDSIR can also be used if D is adapted. Instead, ADSIR is suitable for selecting and using the gain-based intervention filter to remove artefacts in low-dose CT. In this first input, CT images then apply dictionary learning, GDSIR or ADSIR. This proposed method solves different problems, including oversmoothing, artefacts, and noise.

Deora et al. [53] developed a new generative adversarial network (GAN) framework for reconstructing CS-MRI. It improves the quality by combining the patch-based GAN discriminator and the structural similarity index loss. The authors aimed to preserve high-frequency information in the reconstructed image and adequate textural data. In a U-Net-based generator architecture, dense and residual connections were integrated to support more direct data transmission and variable network length. The authors showed that the proposed method performs well compared to other techniques in aspects of reconstruction efficiency and reliability to noise.

Jiang et al. [54] developed a method that retrieves high-resolution CT images from low-resolution ones using a modern semisupervised adversarial generative network technique. Constructing the generator and creating a discriminator based on a supervised system uses a deep unattended network of 16 residual blocks in that paper. A parallel 191 convolution operation is also implemented to minimize the dimensionality of the performance of each hidden layer. The authors performed an objective and subjective systematic study of many standard methods as regards experiments. The analysis results indicate that the proposed network is more robust in the reconstruction of images with a superresolution.

In Table 5, we summarize the papers based on GAN, which are employed in a variety of applications such as image reconstruction, segmentation, and noise reduction.

### 3.7. Deep Learning

Deep learning is an extension of the neural network or ML technique that learns features and tasks directly from data. Data can be images, text, or sound. DL is the sophisticated algorithm of high-performance GPUs due to lots of datasets everywhere. These days, the deep learning algorithm is getting a lot of attention to solve different medical imaging issues such as image reconstruction, segmentation, superresolution, and classification. Deep learning approaches using iterative neural networks and cascaded neural networks have been reported to obtain state-of-the-art results concerning many quantitative quality measures such as PSNR, NRMSE, and SSIM across different imaging modalities. DL-based approaches have been successfully implemented for many applications like image reconstruction, denoising, segmentation, classification, and other image processing applications.

#### 3.7.1. A Survey of Deep Learning

Alder et al. [60] partially learned approach to solving an ill-posed problem. This approach is based on a system of gradient descents. Deep learning is done while using the inverse problem with prior knowledge, providing an increase of 5.4 dB in PSNR over the overall reconstruction of variance. Choosing the error function is not that good, extending it to another iterative scheme.

Kang et al. [61] developed a novel low-dose X-ray CT technique based on DL approach. A novel CNN architecture optimized to denoise CT was proposed to identify and eliminate unique noise patterns from CT. This proposed network divided the work into three parts: (a) counterlet transform can effectively evaporate the directional component of noise to allow better training of deep network; (b) low-dose CT contains complicated noise and to remove such noise CNN has huge prospective; (c) a DNN is suitable for collecting different kinds of data from the large quantity of data. Moreover, the reconstruction time is now much better than those of the existing methods of MBIR.

Wei et al. [62] proposed a joint reconstruction and segmentation method (JRSM) for limited-angle CT scans, which is directly performed on projection data. In their paper, the primal-dual hybrid gradient approach is modified for nonconvex piecewise constant Mumford-Shah (PCMS) model used for discrete value segmentation. The Mumford-Shah model consists of minimization of energy function:

(1) $$\begin{array}{c} \min\Omega \left(u-I\right)2dx+\Omega SI\left|\Delta u\right|2dx+ϒ\left|SI\right|. \end{array}$$

The following optimization problem can be written as a PCMS model:

(2) $$\begin{array}{c} \min i=1Kϒ\left|\partial \Omega \;i\right|+\Omega i\left(u-ci\right)2. \end{array}$$

This paper proposes a JRSM combined with TV-based regularization for CT imaging. The reconstruction with segmentation is much more stable and effective than the alternate approach. But this proposed algorithm is time-consuming.

In Huang et al.'s work [63], for the first time in the thesis, DL is implemented for restricted-angle reconstruction in TXMs. Furthermore, the training of a DNN from synthetic data is being explored for adequate real data in training. In general, U-Net, the standard biomedical imaging CNN, is trained to minimize artefacts in FBP image reconstruction from simulated ellipsoid data and multiattribute data. The proposed technique is tested in 100 ranges of limited-angle tomography on simulated and real data. The proposed approach significantly increases the 3D visualization of the subcellular structures in the *Chlorella* cell for real test results, suggesting its significant importance in the biology, nanoscience, and materials sciences for nanoscale imaging.

To learn a customized scanning strategy, Shen et al. [64] recommended using Reinforcement Learning (RL) to choose the angle and the dose at every desired angle for every different subject. Firstly, the authors formulated CT scanning as an MDP and used a modern in-depth RL approach to overcome that. The CT scanning process was conceived as a Markov decision process, and this was solved using the PPO algorithm. After training on 250 real 2D CT images, the learned custom scanning strategy was validated for 350 CT images. More validation showed that the custom scanning policy led to better overall PSNR reconstruction performance, and it was generalized well to be combined with different reconstruction algorithms. It also showed that the adaptive strategy could change its selection angle and dose assignments to suit other subject areas. One limitation of the proposed approach is the long training period (about 24 hours), even for 2D images, since RL algorithms usually require lots of simulation samples to converge. Furthermore, calculating the reward in our formulated MDP requires running a reconstruction algorithm at every stage. Therefore, it could restrict the application of our system to 3D cases.

Wang et al. [65] first used the SART approach for the restricted-angle TCT projection data. After that, the image reconstructed by the SART approach was imported to a well-trained CNN to remove the artefacts and retain the structures to achieve an improved reconstructed image. Then, the authors used the restricted-angle TCT scanning method and introduced a TCT image reconstruction algorithm based on deep learning. Experimental findings indicate that the proposed technique's performance is good compared to the FBP approach into TCT scanning mode with a limited angle. The proposed method is also efficient in suppressing noise and limited-angle artefacts, maintaining the image structures. The critical issue with the proposed technique is that it requires a large training dataset, and it requires a powerful computer.

Deep learning has been widely applied to biomedical image processing in various applications, as indicated in Table 6.

**Figure 5:**
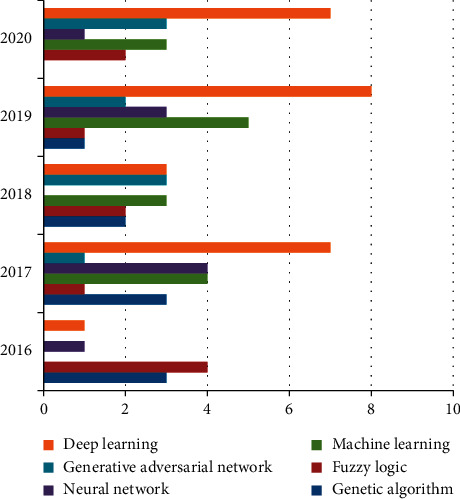
Comparison of soft computing approaches, that is, genetic algorithm, machine learning, neural network, generative adversarial network, and deep learning.

After studying and comparing the application of each approach, different techniques/methods employed, different imaging modalities, a system used, and parameter evaluated, we surveyed all the soft computing techniques in detail, as shown in Tables 1–6. A comparison of all soft computing methods is shown in Figure 5, which includes genetic algorithms, machine learning, neural networks, generative adversarial networks, and deep learning. This comparison reveals that papers based on deep learning algorithms are being published at an increasing rate to address a variety of difficulties in the areas of medical imaging.

## 4. Conclusion

A short review of fuzzy logic, genetic algorithm, neural network, machine learning, generative adversarial network, and deep learning has been discussed in this paper. Also, we studied and compared the application of each approach, different technique/method used, various imaging modalities, system used, and parameter evaluated. After overcoming all the techniques, we discovered that the deep learning algorithm is getting a lot of attention these days to solve several medical imaging issues. In the medical imaging world, these properties have attracted the attention of researchers. We have seen rapid adoption in many conventional and novel applications, such as image reconstruction, segmentation, detection, and classification. Biomedical researchers can take advantage of this survey for inspiration in future CT and PET research. In the coming years, DL with image input is predicted to be the standard in medical imaging technology.

## Figures and Tables

**Table 1:** Overview of papers for biomedical image applications using genetic algorithm methods.

Sr. no.	Reference	Techniques/methods	Imaging modality	Applications	Software used/system used	Parameter evaluation
1	Lihue [10]	GA	ECT	Image reconstruction	MATLAB	Correlation coefficient, image error
2	Qureshi et al. [15]	GA	Transmission tomography	Reconstruction	MATLAB	PSNR, RMSE, EE
3	Miller et al. 2011 [16]	GA	CT	Image reconstruction	MATLAB	MSE
4	Gouicem et al. [11]	GA, fuzzy inference	CT	Image reconstruction	MATLAB	SNR, MSE
5	DCruz et al. 2015 [12]	GA and BNN	Lung CT images	Classification	___	___
6	Chandra et al. [17]	GA	MRI brain tumor detection	Image segmentation	—	SNR, segmentation accuracy
7	Liu et al. [13]	Genetic algorithm (GA), CNN	CT	Image denoising	TensorFlow platform with GeForce GTX TITAN GPUs	PSNR
8	Bahadure et al. [14]	GA & FCM (fuzzy clustering means)	MRI	Segmentation and classification	MATLAB (R2011a)	Accuracy, avg dice coefficient, specificity, sensitivity
9	Kodali and Deb [18]	GA	Ultrasonic tomography (UT)	Reconstruction	SUNE250 machine with 400 Mhz dual-processor using MATLAB 7	Avg. reconstruction times

**Table 2:** Overview of papers for biomedical image applications using fuzzy logic methods.

Sr. no.	Reference	Techniques/methods	Imaging modality	Applications	Software used/system used	Parameter evaluation
1	Mondal and Rajan [20]	Fuzzy logic	PET	Image reconstruction	MATLAB	Residual error, log-likelihood test
2	Sowmya and Raniet [25]	Fuzzy c-means (FCM), competitive neural network	Colour satellite, aerial images	Segmentation	MATLAB 7	MAE, PSNR
3	Devi et al. [26]	NLM fuzzy CD	CT	Image denoising	MATLAB 2018b	PSNR, CP, MSSIM, RMSE
4	Anand [27]	Fuzzy logic	Grayscale images	Noise reduction	MATLAB	PSNR, MSE, execution time
5	Debas et al. [22]	Fuzzy Inference System (FIS), GA	ECT	Image reconstruction	MATLAB	Sensitivity matrix
6	Nithila and Kumar [28]	FCM	CT	Segmentation	MATLAB	Error rate, similarity measure, RMSE
7	Bose [21]	FCM, artificial bee colony	MRI, images, grayscale images, synthetic images	Image segmentation	MATLAB R2012b	
8	Kala and Deepa [23]	Fuzzy logic	MRI	Denoising	MATLAB 2013	PSNR, RMSE, SSIM
9	Ghasemi et al. [24]	Fuzzy dictionary learning	MRI	Image classification	MATLAB (R2017b) on window 10	Accuracy, sensitivity, specificity, convergence speed

**Table 3:** Overview of papers for biomedical image applications using machine learning methods.

Sr. no.	Reference	Techniques/methods	Imaging modality	Applications	Software used/system used	Parameter evaluation
1	Pelt and Batenburg [31]	ML, ANN	CT	Image reconstruction	Python 2.7.3 & NumPy 1.6.3	Mean absolute error
2	Li et al. [32]	ML	CT	Image reconstruction	For training and testing GTX TITAN GPU graphic processor	SSIM, RMSE, PSNR
3	Zheng et al. [34]	Transform learning	LDCT	Image reconstruction	__	RMSE, SSIM, ROI
4	Lee et al. [33]	ML	CT	Image reconstruction	MATLAB 2018a, Keras with a TensorFlow backend, and 4 TITAN-X GPUs	Test accuracy
5	Liu et al. [35]	ML/AI	CT	Image reconstruction and segmentation	Python 3.5.2	Correlation coefficient, mean absolute error (MAE), and dice similarity coefficient (DSC)
6	Kang [36]	ML/transfer learning	MRI	Classification	Python	Accuracy
7	An-Liu [37]	ML	PET/CT	Image segmentation	Python, TITAN-X GPU	Jaccard similarity, accuracy
8	Roth [38]	ML	Multiorgan images	Image segmentation	NVIDIA Clara train SDK	Dice score

**Table 4:** Overview of papers for biomedical image applications using neural network methods.

Sr. no.	Reference	Techniques/methods	Imaging modality	Applications	Software used/system used	Parameter evaluation
1	Srinivasan et al. [43]	Hopfield neural network	CT	Image reconstruction	MATLAB	SNR
2	Cierniak [44]	Hopfield-type neural network	CT	Image reconstruction	—	MSE, SNR
3	Cierniak [45]	Recurrent neural network	CT	Image reconstruction	MATLAB	MSE
4	Chen et al. [46]	Residual encoder-decoder convolution neural network	CT	Denoising	MATLAB 2015b and training was performed on GTX 1080	RMSE, PSNR, SSIM
5	Wu et al. [47]	Artificial neural network (ANN)	CT	Image reconstruction	TensorFlow	SSIM, CNR (contrast to noise ratio)
6	Kartheeswarn [40]	PSO (particle swarm optimization), ANN	CT	Image reconstruction	MATLAB R2010a Parallel computing toolbox (PCI)	MSE, PSNR
7	Chen [48]	Convolution neural network	CT	Noise reduction	MATLAB 2015b, GTX 980 Ti graphics card	PSNR, RMSE, SSIM
8	Souza et al. [41]	CNN	Chest X-ray (CXR)	Lung segmentation and reconstruction	NVIDIA GeForce GTX 1080 Ti graphics card	Average sensitivity, specificity, accuracy, and dice coefficient
9	Chan et al. [42]	Iterative neural Network (INN), DL	CT	MBIR	MATLAB	RMSE, PSNR
10	Wu et al. [49]	CNN	CT	Image reconstruction	Realized NN with TensorFlow & reconstruction with CUDA 9.2	RMSE, SSIM

**Table 5:** Overview of papers for biomedical image applications using generative adversarial network methods.

Sr. no.	Reference	Techniques/methods	Imaging modality	Applications	Software used/system used	Parameter evaluation
1	Wolterink et al. [55]	Generative adversarial network (GAN)	CT	Noise reduction	NVIDIA TITAN-X GPU, 12 GB RAM	PSNR, standard deviation ROI (HU)
2	Pathak et al. [52]	GAN with Wasserstein distance	CT	Denoising	Python with TensorFlow library, NVIDIA TITAN Xp GPU	PSNR, SSIM, MSE
3	Mardani et al. [56]	GAN, CNN, DL	MRI	Image reconstruction	TensorFlow NVIDIA TITAN Xp GPU, 12 GB RAM	SNR, SSIM, reconstruction time (sec)
4	You et al. [57]	GAN, DL, residual learning	CT	Noise reduction	TensorFlow library, NVIDIA TITAN Xp GPU	PSNR, SSIM
5	Hussain et al. (2019) [58]	GAN	MRI	Reconstruction	-	MSE, PSNR
6	Mri et al. [53]	GAN	Compressive sensing MRI	Image reconstruction	Implement using Karras framework, NVIDIA	PSNR, MSSIM
7	Dang et al. 2020 [59]	GAN	CT	Image reconstruction	Karras framework with TensorFlow and training with 4 NVIDIA TITAN-X GPUs	Generator loss, average loss, real and fake score
8	Jiang, 2020 [54]	GAN, residual blocks	CT	Image reconstruction	64-bit Ubuntu OS, TensorFlow V1.2, CUDA tool development kit V8.0, and Python 3.1	Inception score (IS), Frechet Inception Distance(FID)

**Table 6:** Overview of papers for biomedical image applications using deep learning methods.

Sr. no.	Reference	Techniques/methods	Imaging modality	Applications	Software used/system used	Parameter evaluation
1	Milletari et al. [66]	CNN (convolution neural network)	MRI	Image segmentation	Python Caffe framework	Average dice coefficient
2	Kumar et al. [67]	Deep transfer learning	X-ray CT, COVID-19	Reconstruction	ResNet152V2, VGG16, DenseNet201	Recall, precision, F-measure, accuracy
3	A deep learning architecture (2017) [68]	CNN	CT	Reconstruction	For training L-BFGS-B optimization	PSNR, SSIM
4	Chen et al. [69]	DL, CNN	CT	Noise reduction	MATLAB 2015b on a PC	PSNR, RMSE, SSIM
5	Alder et al. [60]	DL	CT	Reconstruction	Python using ODL & TensorFlow	PSNR, runtime
6	Mccann et al. [70]	DCNN	CT, MRI	Image reconstruction	CNN performed on GPU CuDNN (NVIDIA and MATLAB U-Net Toolbox)	SNR
7	Moesdkopset al. [71]	DL, CNN	Brain MRI, breast MRI	Image segmentation		Dice coefficient
8	Kang et al. [61]	DL, CNN	CT	Reconstruction	MatConvNet on MATLAB	PSNR, NRMSE, MSE, RMSE
9	Gu [72]	Deep residual learning	CT	Reconstruction	MATLAB R2015a	PSNR, NRMSE, SSIM
10	Liuet al. [73]	DCNN	CT, MRI	Image reconstruction	MATLAB 2014a and NVIDIA GeForce GTX 1080 GPU	PSNR, SSIM
11	Gupta [74]	CNN projected gradient descent (PGD)	CT	Image reconstruction	MATLAB	SNR, SSIM
12	Jeelani [75]	CNN	MRI	Image reconstruction		PSNR, SSIM
13	Iterative PET image reconstruction using CNN (2018)	CNN	PET	Reconstruction		
14	Kim [76]	CNN	CT	Reconstruction	Trained using TensorFlow on NVIDIA GeForce GTX 1080 Ti	PSNR, SSIM
15	Chen and Davies [77]	CNN	CT	Image superresolution	Implemented in PyTorch and trained on NVIDIA 2080 Ti GPUs	PSNR, GE
16	Aggarwal and Jacob [78]	CNN	MRI	Reconstruction		PSNR, SSIM
17	Chenet al. [42]	INN (iterative neural network), deep learning	CT	Image reconstruction	MATLAB, training on PyTorch	RMSE, PSNR, ROI
18	Wei et al. [62]	DNN	CS-MRI	Segmentation and reconstruction		PSNR, NRMSE
19	Priyanka Wang [79]	Fully symmetric convolution network	Noisy image	Denoising	MATLAB (R2015b) and NVIDIA CuDNN 5.1 deep learning library	PSNR
20	Ding [80]	DNN	X-ray CT	Image reconstruction	PyTorch & trained on NVIDIA TITAN GPUs	PSNR, RMSE, SSIM
21	Gohodratiet al. [81]	DL residual NN, CNN	MRI	Cardiac image reconstruction	TensorFlow on windows, NVIDIA TITAN Xp	SNR, SSIM
22	Huang et al. [63]	DL	X-ray microscopy	Image reconstruction	Adam optimizer	RMSE, SSIM
23	Kofler et al. [82]	CNN	MRI, CT	Image reconstruction	Adam optimizer, ODL library GPU	NRMSE, PSNR, SSIM
25	Whitely et al. [83]	CNN	PET	Image reconstruction	Implemented in PyTorch version 1.3 and trained on NVIDIA TITAN RTX GPU	MAE, SNR
26	Shen et al. [64]	Deep reinforcement learning	CT	Measure angle selection and dose allocation		PSNR, SSIM
27	Chen et al. [84]	DNN	CT	Noise reduction	MATLAB	PSNR, RMSE, SSIM
28	Wang et al. [85]	DL, CNN	Transactional computed tomography (TCT)	Image reconstruction	MATLAB R2016b on PC, NVIDIA GTX 1080, 8 GB RAM	PSNR, MSE, SSIM

## Data Availability

All the data are shared in the main manuscript.
